# Numerical study of the shape parameter dependence of the local radial point interpolation method in linear elasticity

**DOI:** 10.1016/j.mex.2016.03.001

**Published:** 2016-03-10

**Authors:** Ahmed Moussaoui, Touria Bouziane

**Affiliations:** Department of physics, Faculty of Sciences, Moulay Ismail University, B.P.11201 Meknes, Morocco

**Keywords:** Local radial point interpolation method (LRPIM), LRPIM, Meshless method, Radial basis function, Linear Elasticity, Rectangular plate, Support domain

## Abstract

The method LRPIM is a Meshless method with properties of simple implementation of the essential boundary conditions and less costly than the moving least squares (MLS) methods. This method is proposed to overcome the singularity associated to polynomial basis by using radial basis functions. In this paper, we will present a study of a 2D problem of an elastic homogenous rectangular plate by using the method LRPIM. Our numerical investigations will concern the influence of different shape parameters on the domain of convergence,accuracy and using the radial basis function of the thin plate spline. It also will presents a comparison between numerical results for different materials and the convergence domain by precising maximum and minimum values as a function of distribution nodes number. The analytical solution of the deflection confirms the numerical results. The essential points in the method are:

• The LRPIM is derived from the local weak form of the equilibrium equations for solving a thin elastic plate.
• The convergence of the LRPIM method depends on number of parameters derived from local weak form and sub-domains.
• The effect of distributions nodes number by varying nature of material and the radial basis function (TPS).

## Highlights

• The LRPIM is derived from the local weak form of the equilibrium equations for solving a thin elastic plate.
• The method LRPIM is used the trial and test functions in the weak form.
• Convergence of the LRPIM depends on number of parameters derived from local weak form and sub-domains.
• The effect of distributions nodes number by varying nature of material and the RBF-TPS.
• The calculated results are compared with the analytical solution of the deflection.

## Method detail

Meshless method has attracted more and more attention from researchers in recent years.It is regarded as a potential numerical method in computational mechanics. Several meshless methods, such as smooth particle hydrodynamics (SPH) method [1], [2], element free Galerkin (EFG) method [3], meshless local Petrov-Galerkin (MLPG) method [4], [5], [6], [7], [8], point interpolation method (PIM) [9], [10], local point interpolation method (LPIM) [11] and local radial point interpolation method (LRPIM) has been proposed by Liu et al. [10], [12], [13]. In LRPIM, the point interpolation using the radial basis function to construct the shape functions which have the delta function property. The radial basis function (RBFs) is thin plate spline (TPS) [14], [15]. Local weak forms are developed using weighted residual method locally from the partial differential equation of linear elasticity of 2D solids. The number of numerical examples will be presented to demonstrate the convergence and accuracy, validity and efficiency of the present methods. The local radial point interpolation method LRPIM is a Meshless method with properties of simple implementation of the essential boundary conditions and with the lower cost than moving least squares (MLS) methods.

This paper deals with the effect of sizing parameter of subdomains on the convergence and accuracy of the methods. Numerical values will be presented to specify the convergence domain by precising maximum and minimum values as a function of distribution nodes number and by using the radial basis functions TPS (thin plate spline). It also will present a comparison with numerical results for different materials. The analytical solution of the deflection confirms the numerical results. The LRPIM method will be developed to solve the problem of a thin elastic homogenous plate. The local weak form and numerical implementation are presented in section 3, numerical example for 2D problem are given in section 4. Then, the paper will end with the results, discussions and finally the conclusions.

## RPIM shape functions in meshless method

${\text{u}}^{\text{h}}(\text{x})$ is composed of two parts: ${\text{P}}_{\text{j}}(\text{x})$Polynomial basis functions and ${\text{R}}_{\text{i}}(\text{x})$ the radial basis functions RBFs [16], [17], [18], [19], [20]:

(1) $${\text{u}}^{\text{h}}\left(\text{x}\right)=\sum_{\text{i}=1}^{\text{n}}{\text{R}}_{\text{i}}{(\text{x})\text{a}}_{\text{i}}+\sum_{\text{j}=1}^{\text{m}}{\text{P}}_{\text{j}}{(\text{x})\text{b}}_{\text{j}}$$

**n** is the number of field nodes in the local support domain and **m** is the number of polynomial terms.

Radial basis is a function of distance **r:**

(2) $$\text{r}=\sqrt{{(\text{x}-{\text{x}}_{\text{i}})}^{2}+{(\text{y}-{\text{y}}_{\text{i}})}^{2}}$$

The above Eq. (1) can be expressed in the matrix form [17]

(3) $${\text{U}}_{1}=\text{R}\times \text{a}+\text{P}\times \text{b}$$

where ${\text{U}}_{1}$ is the vector of function values: ${\text{U}}_{1}={\left[u_{1},\;u_{2},\;u_{3},\ldots ,\;u_{n}\right]}^{T}$

**R** The moment matrix of RBFs, **P** is the moment matrix of Polynomial basis function and **a, b** are the values of unknowns coefficients (Radial and Polynomial).

We note that, to obtain the unique solutions of Eq. (2), the constraint conditions should be applied as follows [21]:

(4) $$\begin{array}{cc} \sum_{\text{i}=1}^{\text{n}}{\text{P}}_{\text{ij}}(\text{x}){\text{a}}_{\text{i}}=0 & j=1,2,\ldots ,m \end{array}$$

the combining of Eqs. (3) and (4) yields a set of equations in the matrix form:

(5) $${\bar{\text{U}}}_{1}=\left[\begin{array}{l} {\text{U}}_{1}\\ 0 \end{array}\right]=\left[\begin{array}{cc} \text{R} & \text{P}\\ {\text{P}}^{\text{T}} & 0 \end{array}\right]\left[\begin{array}{l} \text{a}\\ \text{b} \end{array}\right]=\text{G}{\text{a}}_{0}$$

The unknown vector in the equation above can be obtained by the inversion of the matrix: $\text{G}=\left[\begin{array}{cc} \text{R} & \text{P}\\ {\text{P}}^{\text{T}} & 0 \end{array}\right]$

Substitution of the vector obtained by inversion of matrix *G* into Eq. (1) leads to:

(6) $${\text{u}}^{\text{h}}\left(\text{x}\right)=\Phi^{\text{T}}\left(\text{x}\right){\text{U}}_{1}=\sum_{\text{i}=1}^{\text{n}}\phi_{\text{i}}{\text{u}}_{\text{i}}$$

## Local weak form method LRPIM

Let us consider a two-dimensional problem of solid mechanics in domain *Ω* bounded by *Г* whose strong-form of governing equation and the essential boundary conditions are given by:

(7) $$\sigma_{\text{ij},\text{j}}(\text{x})+{\text{b}}_{\text{i}}(\text{x})=0$$

(8) $$\sigma_{\text{ij}}{\text{n}}_{\text{j}}=t_{\text{i}}^{0}\;on\;\Gamma_{t}$$

(9) $${\text{u}}_{\text{i}}={\text{u}}_{\text{i}}^{0}\;on\;\Gamma_{u}$$

where in *Ω*: $\sigma^{\text{T}}=[\sigma_{\text{xx}},\sigma_{\text{yy}},\tau_{\text{xy}}]$ is the stress vector, $b^{\text{T}}=[b_{\text{x}},b_{\text{y}}]$ the body force vector. $n=({\text{n}}_{1},{\text{n}}_{2})$ is the vector of unit outward normal at a point on the natural boundaries.

${\text{t}}^{0}$is the prescribed effort, $\left[{\text{u}}_{1},{\text{u}}_{2}\right]$ are the displacement components in the plan and $\left[{\text{u}}_{1}^{0},{\text{u}}_{2}^{0}\right]$ are the prescribed displacement on the essential boundaries.

**Fig. 1:**
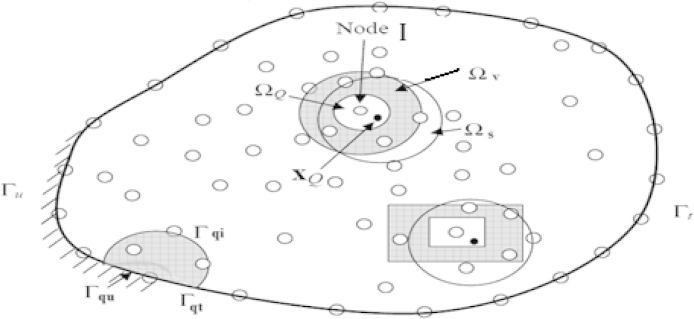
The local sub-domains around point ${\text{x}}_{Q}$ and boundaries.

In the local Petrov-Galerkin approache [3], one may use a weak form over *Ω*_Q_ a local quadrature domain (for node I), which may have an arbitrary shape, and contain the point ${\text{x}}_{\text{Q}}$ in question, see Fig. 1. The generalized local weak form of the differential Eq. (7) is obtained by:

(10) ∫ΩQ(σij,j(x)+b(x)i)υIdΩ=0

where *Ω*_Q_ is the local domain of quadrature for node I and $\upsilon_{\text{I}}$ is the weight or test function ($\upsilon_{I}\in C^{K}(\Omega )$) [4].

Generally, in meshfree methods, the representation of field nodes in the domain will be associated to other repartitions of problem domain: *Ω*_I_ is the influence domain for nodes interpolation, *Ω*_S_ is the support domain for accuracy. For each node *Ω*_υ_ is the weight function domain and *Ω*_Q_ is the quadrature domain for local integration.

Using the divergence theorem [4] in Eq. (10), we obtain:

(11) $$\int_{\Gamma_{Q}}\sigma_{ij}n_{j}\upsilon_{I}d\Gamma -\int_{\Omega_{Q}}\sigma_{ij}\upsilon_{I,j}d\Omega +\int_{\Omega_{Q}}b_{i}\upsilon_{I}d\Omega =0$$

where $\Gamma_{\text{Q}}=\Gamma_{\text{Qi}}\cup \Gamma_{\text{Qu}}\cup \Gamma_{\text{Qt}}$

$\Gamma_{\text{Qi}}$: The internal boundary of the quadrature domain

$\Gamma_{\text{Qt}}$: The part of the natural boundary that intersects with the quadrature domain

$\Gamma_{\text{Qu}}$: The part of the essential boundary that intersects with the quadrature domain

We can then change the expression of Eq. (11):

(12) $$\int_{\Gamma_{\text{Qi}}}\sigma_{\text{ij}}{\text{n}}_{\text{j}}\upsilon_{\text{I}}\text{d}\Gamma +\int_{\Gamma_{\text{Qu}}}\sigma_{\text{ij}}{\text{n}}_{\text{j}}\upsilon_{\text{I}}\text{d}\Gamma +\int_{\Gamma_{\text{Qt}}}\sigma_{\text{ij}}{\text{n}}_{\text{j}}\upsilon_{\text{I}}\text{d}\Gamma -\int_{\Omega_{\text{Q}}}\sigma_{\text{i}\text{j}}\upsilon_{\text{I},\text{j}}\text{d}\Omega +\int_{\Omega_{\text{Q}}}b_{\text{i}}\upsilon_{\text{I}}\text{d}\Omega =0$$

Using the radial point interpolation method (RPIM) shape functions (see sub-section 2), we can approximate the trial function for the displacement at a point x ($\forall \text{x}\in \Omega_{\text{S}}$) as Eq. (6)

The stress vector is defined by:

(13) $$\sigma =C\epsilon =CL_{d}u^{\text{h}}$$

where **C** is the symmetric elasticity tensor of the material

$$C=\left(\begin{array}{ccc} \text{E}/(1-\nu^{2}) & \nu \text{E}/(1-\nu^{2}) & 0\\ \nu \text{E}/(1-\nu^{2}) & \text{E}/(1-\nu^{2}) & 0\\ 0 & 0 & \text{E}/2(1+\nu ) \end{array}\right)$$

Eq. (12) can be written:

(14) $$\int_{\Omega_{Q}}{\bar{v}}_{|}^{T}\sigma d\Omega -\int_{\Gamma_{Qt}}tV_{|}d\Gamma -\int_{\Gamma_{Qu}}tV_{|}d\Gamma =\int_{\Gamma_{Qt}}t^{0}V_{|}d\Gamma +\int_{\Omega_{Q}}V_{|}bd\Omega$$

where ${\bar{V}}_{\text{I}}=\left(\begin{array}{cc} \upsilon_{\text{I},\text{x}} & 0\\ 0 & \upsilon_{\text{I},\text{y}}\\ \upsilon_{\text{I},\text{y}} & \upsilon_{\text{I},\text{x}} \end{array}\right)$ is a matrix that contains the derivatives of the weight functions and $V=\left(\begin{array}{cc} \upsilon_{\text{I}} & 0\\ 0 & \upsilon_{\text{I}} \end{array}\right)$ is the matrix of weight function.

Substituting the differential operator $L_{d}=\left(\begin{array}{cc} \partial /\partial \text{x} & 0\\ 0 & \partial /\partial \text{y}\\ \partial /\partial \text{y} & \partial /\partial \text{x} \end{array}\right)$ into Eq. (13) we obtain:

(15) $$\sigma =C\sum_{\text{I}=1}^{{\text{n}}_{\text{t}}}B_{\text{I}}u_{\text{I}}$$

where $B_{\text{I}}=\left(\begin{array}{cc} \Phi_{\text{I},\text{x}} & 0\\ 0 & \Phi_{\text{I},\text{y}}\\ \Phi_{\text{I},\text{y}} & \Phi_{\text{I},\text{x}} \end{array}\right)$. By using the matrix $L_{\text{n}}=\left(\begin{array}{cc} {\text{n}}_{1} & 0\\ 0 & {\text{n}}_{2}\\ {\text{n}}_{2} & {\text{n}}_{1} \end{array}\right)$, the tractions of a point x can be written as:

(16) $$t=L_{\text{n}}^{\text{T}}\sigma$$

Substituting Eqs. (15) and (16) into Eq. (14), we obtain the discret systems of linear equations for the node I.

$\overset{n_{t}}{\underset{I=1}{\Sigma }}[\int_{\Omega_{Q}}{\bar{V}}_{I}^{T}{CB}_{|}d\Omega -\int_{\Gamma_{Qt}}L_{n}^{T}{CB}_{|}V_{I}d\Gamma -\int_{\Gamma_{Qu}}L_{n}^{T}{CB}_{|}V_{I}d\Gamma ]u_{|}=\int_{\Gamma_{it}}t^{0}V_{I}d\Gamma +\int_{\Omega Q}V_{I}bd\Omega$ (17)

The matrix form of Eq. (17) can be written as in matrix form:

(18) $$\sum_{\text{I}=1}^{{\text{n}}_{\text{t}}}K_{\text{I}}u_{\text{I}}=f_{\text{I}}$$

where **K_I_** is a 2 × 2 matrix called a nodal stiffness matrix, given by

(19) $$K_{|}=\int_{\Omega_{Q}}{\bar{V}}_{I}^{T}{CB}_{|}d\Omega -\int_{\Gamma_{Qt}}L_{n}^{T}{CB}_{|}V_{I}d\Gamma -\int_{\Gamma_{Qu}}L_{n}^{T}{CB}_{|}V_{I}d\Gamma$$

and nodal force vector with contributions from body forces applied in the problem domains:

(20) $$f_{\text{I}}=\int_{\Gamma_{\text{Qi}}}{\text{t}}^{0}V_{\text{I}}\text{d}\Gamma +\int_{\Omega_{\text{Q}}}V_{\text{I}}b\text{d}\Omega$$

where n_0_ denote the set of the nodes in the support domain $\Omega_{\text{S}}$ of point ${\text{x}}_{\text{Q}}$.

Two independent linear equations can be obtained for each node in the entire problem domain and by assembling all these n × 2 equations to obtain the final global system equations:

(21) $$k_{2\text{n}\times 2t_{}}u_{2{\text{n}}_{}\times 1}=f_{2\text{n}\times 1}$$

To solve the precedent system, the standard Gauss quadrature formula is applied with 16 G points [3], [22] for calculating integrals in Eqs. (19) and (20) on both boundary and domain.

The size of quadrature domain is specified by setting $\alpha_{\text{Q}}=2$ and a regular distribution of nodes on the mid-surface of plate in (x, y) plane is employed.

## Numerical 2D elastostatic example

**Fig. 2:**
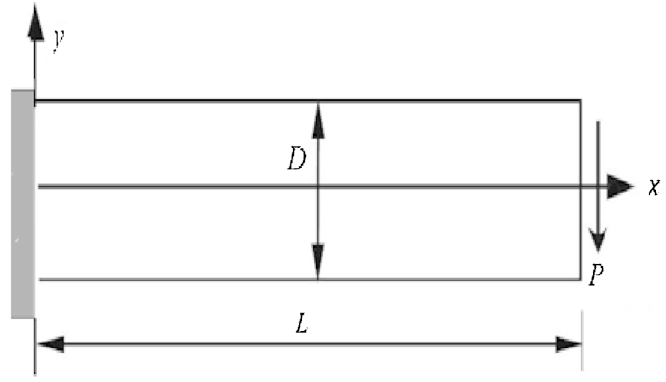
Cantilever plate subjected to distributed traction at the free end.

This section is about numerical results for a cantilever rectangular plate see (Fig. 2). First, the effects of the size of support and quadrature domains were investigated and the convergence of LRPIM method for several materials was examined numerically; then, comparisons will be made with the analytic solution for several materials [23] We choose: steel, zinc, aluminium and copper with: ($\text{E}=3.10^{7}\text{N}/{\text{m}}^{2}$, ν=0.3; $\text{E}=113.10^{5}\text{N}/{\text{m}}^{2}$, ν=0.25; $\text{E}=1.10^{7}\text{N}/{\text{m}}^{2}$, ν=0.34; $\text{E}=17.10^{6}\text{N}/{\text{m}}^{2}$, ν=0.33) respectively. Dimensions of the plate are denoted: height: D=12m, length: L=48m, the thickness: unit and finally for Loading: $\text{P}=10^{3}\text{N}$

In our numerical calculations many regular distributions of nodes were considered **n_t_**: 18, 55, 91, 175 and 189. To calculate the error energy, a background cells are required; then, for each value of **n_t_** the number of cell was varied. To obtain numerical values, the distribution of the deflection through the plates, size of support domain is varied and $\alpha_{\text{Q}}$ is the size of the quadrature domain and fixed to the value 2.

**Fig. 3:**
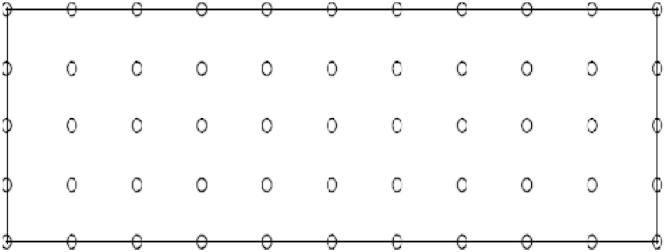
Regular field nodes distribution on the problem domain and boundaries.

The sizes of support domain $\Omega_{s}$(quadrature domain $\Omega_{Q}$ resp.) are defined by: ${\text{d}}_{\text{s}}=\alpha_{\text{s}}{\text{d}}_{\text{c}}$ (${\text{r}}_{\text{Q}}=\alpha_{\text{Q}}{\text{d}}_{\text{c}\text{I}}$resp.) where ${\text{d}}_{\text{c}}$(${\text{d}}_{\text{c}\text{I}}$ resp) is the nodal spacing near node I (see Fig. 3) and $\alpha_{\text{s}}$($\alpha_{\text{Q}}$ resp) is the size of the support domain $\Omega_{s}$ (local quadrature domains resp) for node I. The sizes of support domain $\Omega_{s}$(quadrature domains resp) will be respectively determined in x and y directions. For simplicity, we put: $\alpha_{\text{Sx}}=\alpha_{\text{Sy}}=\alpha_{\text{S}}$($\alpha_{\text{Qx}}=\alpha_{\text{Qy}}=\alpha_{\text{Q}}$resp) are used for $\Omega_{s}$($\Omega_{Q}$ resp).

## Results and discussions

The standard Gaussian quadrature formula with 16 G points and the radial point interpolation method (RPIM) approximation, linear polynomial basis functions are applied. The cubic or quadratic spline functions a used as the test functions in the LRPIM local weak-form.

Throughout this section and for all calculations $\alpha_{\text{Q}}=2$ was fixed.

### Simulation results and comparison with the radial basis function RBF-TPS (of a thin plate spline)

The use of radial basis function RBF-TPS have not been extensively studied on literature. We give in this paragraph numerical results for different materials.

**Fig. 4:**
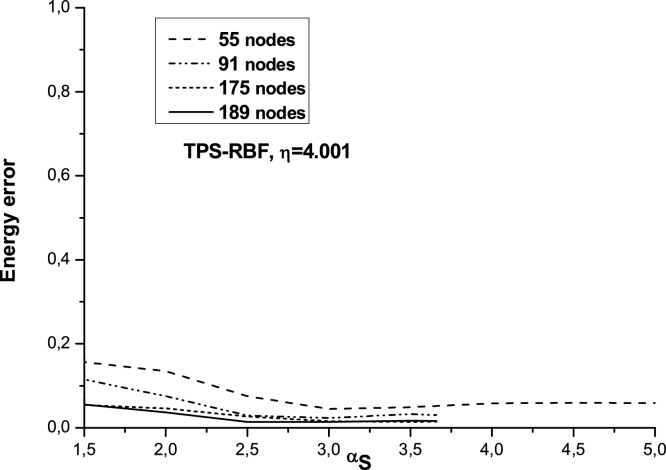
Influence of the $\alpha_{\text{S}}$ size of $\Omega_{\text{s}}$ on energy error for different distribution nodes numbers (steel).

Fig. 4 shows the energy error as a function of $\alpha_{\text{S}}$ for cubic spline $\upsilon_{1}$ used as the test function and for the chosen radial basis RBF-TBS and steel. The results which are calculated by the LRPIM method are influenced by different parameters. This method shows the variation of maximum and minimum values of $\alpha_{\text{S}}$ of convergence domain by increasing the distribution regular field number nodes **n_t_**: = 55, 91, 175,189. It can be seen from Fig. 4 that if the values of $\alpha_{\text{S}}$ are smaller than 1.80, the energy error is large and LRPIM method is not convergent. The domain of convergence reaches the maximum value at $\alpha_{\text{S}}=5$ for if **n_t_ **= 55.

For **n_t_** = 91, 175 or 189 the domain convergence is smaller than that obtained with **n_t_** = 55. The greater extremity value of the convergence domain is now $\alpha_{\text{S}}$ equal to 3.66 for (**n_t_** = 91, 175, 189). The convergence domain is noticed between a small value $\alpha_{\text{S}}=1.80$ and the greater value $\alpha_{\text{S}}=5$.

**Fig. 5:**
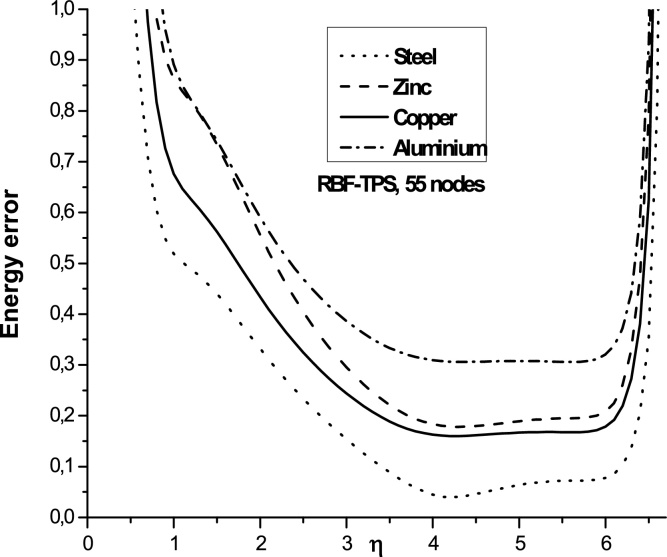
Variation of the energy error as a function of η for different materials, ${\text{n}}_{\text{t}}=55$and $\alpha_{\text{S}}=3$.

**Fig. 6:**
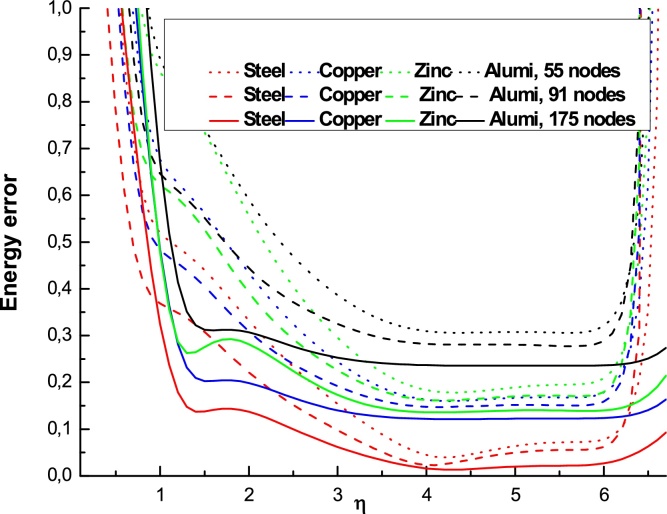
Variation of the energy error as a function of ηfor different materials, **n_t_** = 55, 91, 175 and $\alpha_{\text{S}}=3$.

**Fig. 7:**
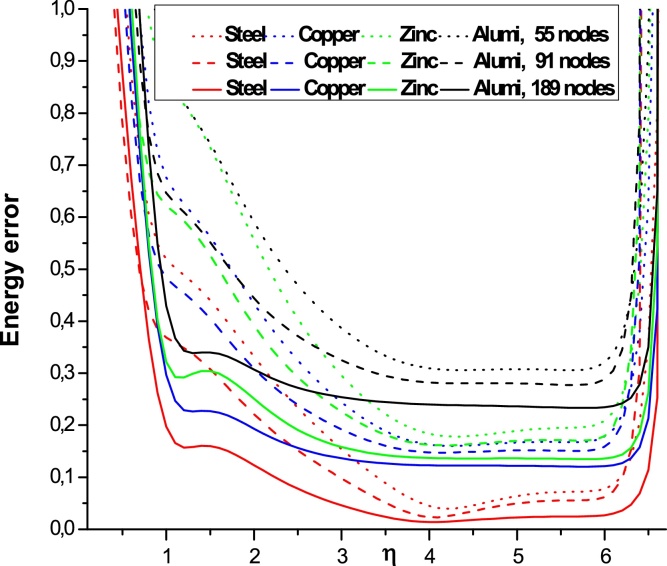
Variation of the energy error as a function of ηfor different materials, **n_t_** = 55, 91, 189 and $\alpha_{\text{S}}=3$.

The Figs. 5–7 show the variation of the error energy as a function of the shape parameter η for different values of *E* and ν (different materials) and for different values of the number **n_t_** = 55, 91, 175, 189 and $\alpha_{\text{S}}=3$. We find that all the curves of different materials have similar paces for a fixed value *η* in the following domains:

For ${\text{n}}_{\text{t}}=55$, the convergence domain is: 3.25<η<6.25

For ${\text{n}}_{\text{t}}=91$, the convergence domain is: 1.5<η<6.25

For ${\text{n}}_{\text{t}}=175$, the convergence domain is: 0.5<η<6.5

For ${\text{n}}_{\text{t}}=189$, the convergence domain is: 1<η<6.5

The results illustrated in the Fig. 5, Fig. 6, Fig. 7 to the number of nodes **n_t_** = 55, 91, 175 and 189 with the radial basis RBF-TPS give the domain of convergence large enough for the different types of materials studied. This shows better convergence than that given in references [10], [24], [25]. These authors give a single value (η=4.001). We show here that the values *η* can be varied in a broader segment depending on the number ${\text{n}}_{\text{t}}$.We found that the domain of convergence is more larger than the value given by Liu et al.

**Fig. 8:**
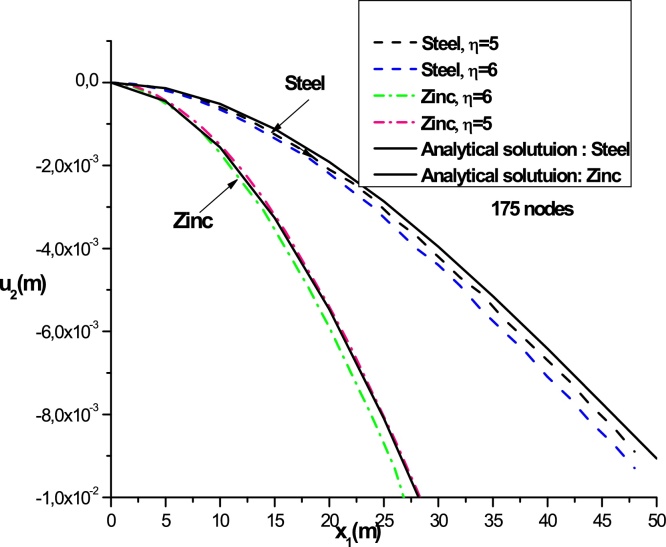
Deflections as a function of ${\text{x}}_{1}$ at ${\text{x}}_{2}=0$ for the radial basis and analytical solution for two materials (steel and zinc, *η* = 5; 6 and ${\text{n}}_{\text{t}}=175$; $\alpha_{\text{S}}=3$).

Fig. 8 shows the variations of displacement as a function of ${\text{x}}_{1}$ for ${\text{x}}_{2}=0$ with the shape parameter *η* (*η* = 5 and 6) of the radial basis function RBF-TPS. We used two studied materials: Steel and Zinc. We note that there is a coincidence between the analytical solution and the result obtained by the LRPIM method of the radial basis RBF-TPS. It shows the convergence of LRPIM method (see Fig. 7)

## Conclusion

In this paper, the meshless LRPIM method is employed for solving a 2D elastostatic problem. The LRPIM method depends on the sizing parameter $\alpha_{\text{S}}$, which is associated to different parameters coming from the weak form formulation. In the study $\alpha_{\text{Q}}=2$ is fixed, the nature of convergence domain as a function of $\alpha_{\text{S}}$ and the effect of distributions nodes number ${\text{n}}_{\text{t}}$ by varying nature of material and the radial basis functions RBF-TPS. We conclude that for small value of ${\text{n}}_{\text{t}}(55)$ lead to the upper extremity of convergence domain which is limited to $\alpha_{\text{S}}=5$. For greater values of $n_{t}$(91, 175, 189), we found that the maximal value for convergence domain equals:$\alpha_{S}=3.66$. No dependency is noted of the maximum extremity value $\alpha_{\text{S}}$ of convergence domain and the elastic nature of materials.

The results obtained for the number of nodes **n_t_** = 55, 91, 175, 189 and for the radial basis RBF-TPS, give a domain of convergence which is large enough for the different types of materials studied. This shows a better convergence more than the result that is found in the references [10], [24], [25]. There is a good agreement with the analytical solution of the deflection.
